# REL-1017 (Esmethadone), A Novel NMDAR Blocker for the Treatment of MDD is Not Neurotoxic in Sprague-Dawley Rats

**DOI:** 10.3389/fphar.2022.863959

**Published:** 2022-04-25

**Authors:** Francesco Bifari, Marco Pappagallo, Michael Bleavins, Sergio Traversa, Franco Folli, Paolo L. Manfredi

**Affiliations:** ^1^ Department of Medical Biotechnology and Translational Medicine, University of Milan, Milan, Italy; ^2^ Relmada Therapeutics, Coral Gables, FL, United States; ^3^ Environmental Health Sciences and School of Public Health, University of Michigan, Ann Arbor, MI, United States; ^4^ Department of Health Sciences, University of Milano, Milan, Italy

**Keywords:** esmethadone, neurotoxic, REL-1017, N-methyl-D-aspartate receptor channel blocker, Olney’s lesion

## Abstract

REL-1017 (esmethadone; dextromethadone; (S)-methadone) is the opioid-inactive dextro-isomer of the racemic mixture, (R, S)-methadone. REL-1017 acts as a low affinity, low potency N-methyl-D-aspartate receptor (NMDAR) channel blocker with rapid, robust, and sustained therapeutic effects in patients with major depressive disorder (MDD). Systemic administration of NMDAR blockers may cause transient and reversible pathomorphological alterations in brain cortical neurons characterized by cytoplasmic vacuolization, which are called Olney’s lesions, and may also lead to irreversible neuronal necrosis. We determined whether REL-1017 administration via oral gavage for 1–4 days to Sprague-Dawley rats could produce Olney’s lesions and cortical neuronal death and microgliosis as compared with MK-801, a known neurotoxic potent NMDAR blocker. As previously reported, MK-801 produced Olney’s lesions, neuronal necrosis and cortical microgliosis, and impaired behavior and activity. In contrast, administration of REL-1017 at low (20–31.25 mg/kg in females and males), medium (40–62.5 mg/kg) or high (80–110 mg/kg) doses did not cause pathomorphological changes in brain neurons and did not cause impaired behavior and activity. In conclusion, REL-1017 did not produce initial or cumulative neurotoxic effects or other evidence of damage to cortical neurons, further encouraging the development of REL-1017 as a potentially safe novel candidate for rapid treatment of MDD.

## Introduction

REL-1017 (esmethadone; dextromethadone; (S)-methadone) is the opioid-inactive dextro-isomer of the racemic mixture, (S, R)-methadone. REL-1017 is devoid of clinically meaningful opioid effects, including dependence and abusability (DEA, 2019; Henningfield et al., 2021). REL-1017 acts as a low affinity, low potency N-methyl-D-aspartate receptor (NMDAR) channel blocker, with half maximal inhibitory concentration (IC_50_) values in the low micromolar range (Gorman et al., 1997; Bettini et al., 2021a). Uncompetitive NMDAR channel blockers are emerging as a new class of clinically well tolerated drugs effective for the treatment of major depressive disorder (MDD) and other neuropsychiatric and neurodegenerative diseases. Memantine and the combination of dextromethorphan and quinidine have been approved by the Food and Drug Administration (FDA) to treat Alzheimer's disease and pseudobulbar affect, respectively. Esketamine has been FDA approved for the treatment of resistant MDD (Kim et al., 2019). REL-1017 demonstrated rapid, robust and sustained therapeutic effects in patients with major depressive disorder (MDD) in a double blind, placebo-controlled Phase 2 study (Fava et al., 2022). Of interest, we demonstrated that esmethadone is a low-affinity, high-trapping, uncompetitive blocker of human NMDARs with preferential binding to GluN1-GluN2D subtypes in the presence of physiological Mg^2+^concentration (Bettini et al., 2021a; Bettini et al., 2021b; Bettini et al., 2021c).

Systemic administration of drugs that block NMDARs has been shown to cause acute pathomorphological changes in cortical neurons of the adult rat brain called Olney’s lesions (Olney et al., 1989). These potentially reversible lesions consist of characteristic cytoplasmic vacuolization, which can be seen by light microscopy in H&E stained brain sections and are particularly evident in neurons of the rat posterior cingulate and retro-splenial cortex (Olney et al., 1989). Higher doses or more prolonged treatment with NMDAR antagonists have been shown to induce irreversible neuronal degeneration (Olney et al., 1991; Fix et al., 1993), including necrosis in the posterior cingulate and retrosplenial cortices, and in other brain regions (Ellison, 1994; Horvath et al., 1997; Wozniak et al., 1998; Newcomer et al., 2000).

This study was designed to determine whether the novel low affinity, low potency NMDAR channel blocker REL-1017 administered once daily via oral gavage for 1–4 days to Sprague-Dawley rats could produce initial or cumulative neurotoxic effects or other evidence of damage, including cortical neuron death and microgliosis. For this purpose, we studied both female and male animals to see if there was a gender difference in the potential REL-1017 neurotoxic effects when compared to the known neurotoxic NMDAR blocker, MK801.

## Methods

### Animals

Sprague-Dawley female and male rats, 10.5 weeks old, weighing between 201 and 374 g (Charles River, Raleigh, North Carolina, United States) were employed in the study. Each animal was identified using a subcutaneously implanted electronic identification chip and randomly assigned to groups. Two to three animals were housed in solid-bottom cages with nonaromatic bedding. The housing was equipped with an automatic watering valve as specified in the USDA Animal Welfare Act (9 Code of Federal Regulations [CFR], Parts 1, 2 and 3) and as described in the Guide for the Care and Use of Laboratory Animals (National Research Council, Current edition). Each cage was clearly labeled with study, group, animal number, and sex. Target temperatures of 68°F–79°F with a target relative humidity of 30–70% were maintained. A 12-h light/12-h dark cycle was maintained. Block Lab Diet (Certified Rodent Diet #5002, PMI Nutrition International, Inc.) was provided ad libitum. Tap water was available ad libitum to each animal *via* an automatic watering system; the drinking water used was monitored for specified contaminants at periodic intervals. All procedures were conducted in accordance with the Institutional Animal Care and Use Committee and the Eighth Edition of the Guide for Care and Use of Laboratory Animals (National Research Council 2017).

### Study Design

**FIGURE 1:**
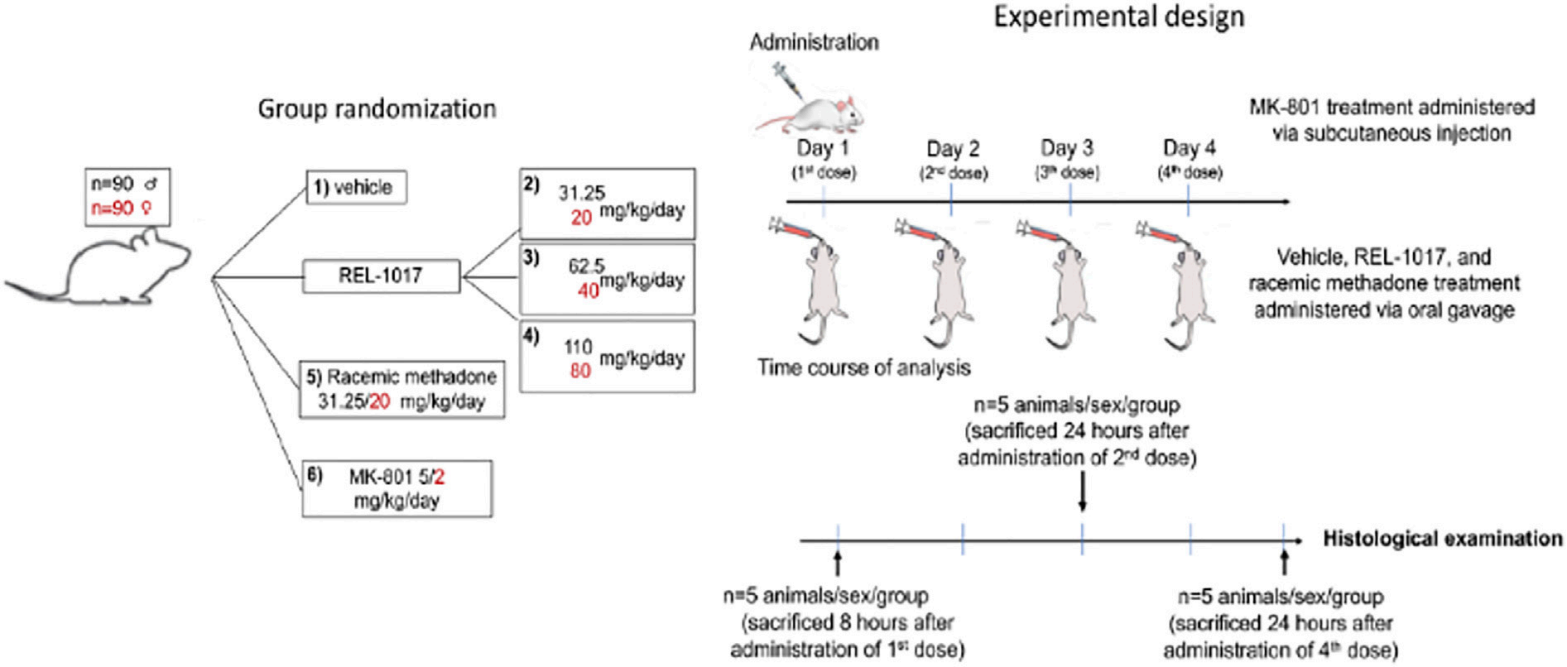
Study design. The vehicle control, REL-1017, and methadone racemate were administered once daily for 4 days during the study *via* oral gavage. The dose levels for male rats receiving the REL-1017 were 31.25, 62.5, and 110 mg/kg/day. Female rats received 20, 40, and 80 mg/kg/day. The dose volume for all groups was 5 ml/kg. The groups receiving the vehicle control or methadone racemate were administered in the same manner as the REL-1017 at a dose level of 0 (vehicle control), 31.25 (males), or 20 (females) mg/kg/day. The positive control was administered once on Day 1 *via* subcutaneous injection at a dose level of 5 (males) or 2 (females) mg/kg at a dose volume of 1 ml/kg. Clinical evaluation was performed daily. The presence of neuronal vacuoles (Olney’s effect) was analyzed by light microscopy in H & E stained brain tissue (2 um thick), on brains obtained 8 h after the administration of the first dose of drugs. Neuronal necrosis and microgliosis were assessed by histological examinations in brains collected 24 h after the administration of the second dose (Day 3 of the study) and 24 h after the administration of the fourth dose (Day 5 of the study).

As described in Figure 1 and Table 1, 90 male and 90 female Sprague-Dawley rats were subdivided into 12 groups of 15 rats each. This study was conducted in compliance with Good Laboratory Practice regulations at Charles River Laboratory (Raleigh, North Carolina, United States). Group 1 animals received vehicle control solution *via* oral gavage (0 mg/kg/day; *n* = 15 males). Groups 2, 3, and 4 animals received REL-1017 *via* oral gavage (31.25, 62.5, and 110 mg/kg/day, respectively; *n* = 15 males/group, total number of animals = 45 males). Group 5 animals received methadone racemate *via* oral gavage (31.25 mg/kg/day; *n* = 15 males). Group 6 animals were administered MK-801 subcutaneously (5 mg/kg/day; *n* = 15 males). Group 7 animals received the vehicle control *via* oral gavage (0 mg/kg/day; *n* = 15 females). Group 8, 9, and 10 animals received REL-1017 *via* oral gavage (20, 40, and 80 mg/kg/day, respectively; *n* = 15 females/group, total number of animals = 45 females). Group 11 animals were administered methadone racemate *via* oral gavage (20 mg/kg/day; *n* = 15 females). Group 12 animals were administered MK-801 subcutaneously (2 mg/kg/day; *n* = 15 females). Groups 1–5 (males) and 7–11 (females) animals were administered experimental articles (REL-1017 or methadone) or vehicle control solutions once a day for 4 days. These doses were selected based on previously conducted rat studies with REL-1017 that determined tolerability and showed that females had substantially higher systemic exposures at a given dose than males. The positive control, MK-801, was administered once on Day 1 *via* subcutaneous injection at a dose level of 5 (males) or 2 (females) mg/kg/day and at a dose volume of 1 ml/kg. The subcutaneous injections were administered between the skin and the underlying layers of tissue in the scapular region of the animal. Individual doses were based on the most recent body weights. Clinical evaluation was performed daily on all animals. Five animals/sex/group were euthanized on Day 1 (8 h post first dose), on Day 3 (24 h after the second dose), and on Day 5 (24 h after the fourth dose). The presence of neuronal vacuoles (Olney’s effect) was assessed by light microscopy in H&E stained brain 2 μm thick sections, with emphasis on the cingulate gyrus. Neuronal necrosis was determined by Fluoro Jade B staining. As an additional indication of drug-induced brain toxicity, microgliosis in the brain cortex was also assessed (see Figure 1 and Table 1).

**TABLE 1:** Experimental design.

Group	Test material	Dose route	Dose level (mg/kg/d)	Dose volume^a^ (ml/kg)	Dose concentration (mg/ml)	Number of animals^b^	
						Males	Females
1	Vehicle control	Oral gavage	0	5	0	15	—
2	REL-1017	Oral gavage	31.25	5	6.25	15	—
3	REL-1017	Oral gavage	62.5	5	12.5	15	—
4	REL-1017	Oral gavage	110	5	22	15	—
5	Methadone racemate	Oral gavage	31.25	5	6.25	15	—
6	MK-801 (positive control)	Subcutaneous	5	1	5	15	—
7	Vehicle control	Oral gavage	0	5	0	—	15
8	REL-1017	Oral gavage	20	5	4	—	15
9	REL-1017	Oral gavage	40	5	8	—	15
10	REL-1017	Oral gavage	80	5	16	—	15
11	Methadone racemate	Oral gavage	20	5	4	—	15
12	MK-801 (positive control)	Subcutaneous	2	1	2	—	15

a Based on the most recent body weight measurement.

b Five animals/sex/group were euthanized on Day 1 (8 h post first dose), on Day 3 (24 h after the second dose), and on Day 5 (24 h after the fourth dose). Designated animals in Groups 6 and 12 were euthanized at approximately the same time as the cohorts in the corresponding REL-1017, groups.

### Clinical Observations

All animals were evaluated daily throughout the study for clinical changes. Observations included evaluation of the skin, fur, eyes, ears, nose, oral cavity, thorax, abdomen, external genitalia, limbs and feet, respiratory, and circulatory effects, autonomic effects such as salivation, nervous system effects including tremors, convulsions, reactivity to handling, and unusual behavior. Rat body weights were measured and recorded before the study (Day 1) and on the day of necropsy (Days 1, 3, and 5).

### Brain Tissue Fixation and Histology

Animals were euthanized by euthanasia solution injection, followed by whole-body intravascular perfusion with 0.9% sodium chloride followed by 4% paraformaldehyde. For histochemical analysis, whole brains were sectioned into 2 μm slices. Brain tissue blocks were embedded in paraffin. Sections were obtained using a 2-micron block advance, slide mounted, and stained with hematoxylin and eosin (H&E). Serial sections were also stained with Fluoro Jade B (FJB), a sensitive and specific fluorescence staining for neuronal necrosis (BSENTR-150-FJB, VWR, Suwanee, GA 30024, United States). FJB staining was evaluated employing a fluorescence microscope with a FITC filter. We performed immunohistochemistry (IHC) analysis for ionic calcium-binding adaptor molecule 1 (IBA-1), a protein expressed in macrophages and microglial cells, which is upregulated when these cells are activated in pathological conditions, making this stain a sensitive indicator of microglial activation in the brain. For IHC, slices were incubated in 0.25%/0.5% Triton X-100, 2% bovine serum albumin (BSA) with a primary antibody (1:600) against rat IBA1 (Wako, Code No. 019-19741) for one hour at room temperature. Antigen detection was performed by polymer methods by using labeled polymer prepared by combining amino acid polymer with multiple molecules of alkaline phosphatase and goat anti-mouse Ig reduced to Fab’. Following incubation with DAB solution (10 mg of 3,3′-diaminobenzidine, tetrahydrochloride 50 ml of 0.05 M Tris-HCl buffer, 15 mM NaN_3_ pH 7.6, 50 μL of 5% H_2_O_2_, 34 mg of imidazole in distilled water), counterstaining with hematoxylin to stain the nuclei was performed.

### Grading of the Histological Findings

All stained brain tissue slices were independently assessed by Tox Path Services, LLC (Frederick, Maryland) neuropathologists. Quantification of the severity of morphological and IHC parameters was assessed by using the following 1 to 5 grade score. 1—Minimal severity grade. A microscopic change that barely exceeds normal limits or a normal condition. Typically, these are microscopic changes that are sporadic, focal, and of no biologic significance to the function or structure of the tissue. Microscopic changes graded as “1” are not readily apparent in the tissue. Generally, a minimal change affects less than 1% of the total tissue that might be possibly affected. 2—Mild severity grade. A microscopic change that is more readily apparent in the tissue than a minimal (Grade 1) change but is still unlikely to produce any structural or functional impairment. Typically, a grade of mild indicates the microscopic change was present in one or a few foci. Generally, a microscopic change graded as mild affects less than 5% of the total tissue that might be possibly affected. 3—Moderate severity grade. A microscopic change that is readily observed in the section, but still of limited severity and unlikely to have any biologically significant effect on the overall structure or function of the tissue. Moderate changes may be focal, multifocal, or even diffuse. 4—Marked severity grade. A microscopic change that is a prominent, conspicuous, and easily identified feature of the tissue, but is still not present at what would be considered to be the maximum possible effect. A marked change would reasonably be expected to have some effect on the structure and/or function of a tissue, although the corresponding functional change may or may not be apparent. 5—Severe severity grade. A microscopic change that is prominent, cconspicuous, and easily identified feature of the tissue, is present at a maximum severity (affects the majority of the tissue and/or is very pronounced in one or more foci), and/or is present at a severity that would be expected to have a prominent effect on the structure and/or function of a tissue, although the correlating functional change may or may not be apparent.

In particular, to evaluate the severity of the Olney effect, we used the following scoring:

**Table:** 

**Vacuolation**	
Severity Grade 1 (minimal)	One or a few foci (or affected cells) present
Severity Grade 2 (mild)	More than a few foci (or affected cells) present
Severity Grade 3 (moderate)	Focally extensive to more diffuse area/groups of cells affected
Severity Grade 4 (marked)	Prominent portion of the tissue and/or cell type affected
Severity Grade 5 (severe)	Greater than 40% of the region/cells affected

To evaluate the severity of the neuronal necrosis, we used the following scoring:

**Table:** 

**Necrosis, Neuronal**	
Severity Grade 1 (minimal)	<1% of neurons in a tissue/region affected
Severity Grade 2 (mild)	1–5% of neurons in a tissue/region affected
Severity Grade 3 (moderate)	5–15% of neurons in a tissue/region affected
Severity Grade 4 (marked)	15–40% of neurons in a tissue/region affected
Severity Grade 5 (severe)	>40% of neurons in a tissue/region affected

To evaluate the severity of the microgliosis, we used the following scoring:

**Table:** 

**Migrogliosis**	
Severity Grade 1 (minimal)	Microgliosis barely exceeds normal limits; it is typically focal and/or sporadic and of no biological relevance; it is a common change in control animals, especially monkeys
Severity Grade 2 (mild)	Microgliosis more readily apparent than minimal severity grade, typically focal or multifocal; typically a level of severity uncommonly encountered in controls
Severity Grade 3 (moderate)	Microgliosis readily apparent; typically focally extensive or multifocal and typically a level of severity uncommonly encountered in controls
Severity Grade 4 (marked)	Microgliosis pronounced, affecting a prominent portion of a given brain/spinal cord region, typically at a level of severity not encountered in controls
Severity Grade 5 (severe)	Microgliosis very pronounced, affecting a prominent portion of a given brain/spinal cord region, typically at a level of severity not encountered in controls

### Statistical Analysis

Statistical analysis was performed with GraphPad Prism 8 software (GraphPad Software, Inc. La Jolla, CA, United States). Data are shown as the mean ± standard deviation (SD) or standard error of the mean (SEM) of the number of measures as indicated. To assess the drug effect at different time-points, the different scores were compared using the two-way analysis of variance (ANOVA), corrected by the Tukey test. *p* ≤ 0.05 was considered statistically significant; *p* > 0.05 was considered not statistically significant (ns).

## Results

The aim of the study was to determine whether the novel low affinity, low potency NMDAR channel blocker REL-1017, administered once daily *via* oral gavage for 1–4 days to male and female Sprague-Dawley rats, induced transient (Olney’s lesions) and irreversible (necrosis) pathomorphological changes to the posterior cingulate and retrosplenial brain cortical neurons. As a positive control, animals were treated with another NMDAR channel blocker, dizocilpine (MK-801) (Horvath et al., 1997) (see Figure 1 and Table 1).

### REL-1017 Did Not Cause Behavioral, External Appearance, nor Meaningful Body Weight Changes

The behavioral effects of MK-801 treatment include a rapid onset of ataxia, which is followed by recumbency (Clineschmidt et al., 1982). In this protocol, clinical evaluation was done daily. In male (5 mg/kg/day) and female (2 mg/kg/day) mice treated with MK-801, we observed clinical findings related to decreased activity (92.5%), impaired limb function (66.7%), ventral and/or lateral recumbency (63.4%), and ataxia (57.5%). None of these clinical parameters were neither statistically nor meaningfully altered in REL-1017 at any dose (low, medium, and high) in either male or females (Table 2a, 2b).

**TABLE 2a:** Clinical observations—female rats.

	REL-1017				Methadone	MK-801	*p*
	Dose (mg/kg/d)						
	0	20	40	80	20	2	
Number of animals	15	15	15	15	15	15	(MK801 *vs.* all)
Behavior/activity							
Activity decreased							
Number of times recorded	0	0	0	0	2	47	—
Number of animals affected	—	—	—	—	2	15	<0.0001
Ataxia							
Number of times recorded	0	0	0	0	0	28	—
Number of animals affected	—	—	—	—	—	10	<0.0001
Lateral recumbency							
Number of times recorded	0	0	0	0	0	3	—
Number of animals affected	—	—	—	—	—	3	<0.05
Salivation							
Number of times recorded	0	0	0	0	0	1	—
Number of animals affected	—	—	—	—	—	1	ns
Ventral recumbency							
Number of times recorded	0	0	0	0	2	20	—
Number of animals affected	—	—	—	—	2	14	<0.0001
External Appearance							
Limb function impaired							
Number of times recorded	0	0	0	0	4	48	—
Number of animals affected	—	—	—	—	1	10	<0.0001
Material around eyes, black							
Number of times recorded	0	0	0	0	0	5	—
Number of animals affected	—	—	—	—	—	3	<0.05
Material around eyes, red							
Number of times recorded	0	0	1	0	0	11	—
Number of animals affected	—	—	1	—	—	4	<0.05
Material around nose, black							
Number of times recorded	0	0	0	0	0	2	—
Number of animals affected	—	—	—	—	—	2	ns
Material around nose, red							
Number of times recorded	0	1	0	1	0	5	—
Number of animals affected	—	1	—	1	—	5	<0.01
Thin							
Number of times recorded	0	1	1	1	1	24	—
Number of animals affected	—	1	1	1	1	8	<0.0001
Pelage/Skin							
Hair wet							
Number of times recorded	0	0	0	0	0	3	—
Number of animals affected	—	—	—	—	—	2	ns
Skin discoloured, red							
Number of times recorded	0	0	0	0	0	5	—
Number of animals affected	—	—	—	—	—	1	ns
Skin discoloured, red/black							
Number of times recorded	0	0	0	0	0	1	—
Number of animals affected	—	—	—	—	—	1	ns
Behaviour/activity							
Righting reflex impaired							
Number of times recorded	0	0	0	0	0	7	—
Number of animals affected	—	—	—	—	—	7	<0.0001

**TABLE 2b:** Clinical observations—Male rats.

	REL-1017				Methadone	MK-801	*p*
	Dose (mg/kg/d)						
	0	20	40	80	20	2	
Number of animals	15	15	15	15	15	15	(MK801 *vs.* all)
Behaviour/activity							
Activity decreased							
Number of times recorded	0	0	0	0	0	40	—
Number of animals affected	—	—	—	—	—	12	<0.0001
Ataxia							
Number of times recorded	0	0	0	0	0	23	—
Number of animals affected	—	—	—	—	—	13	<0.0001
Salivation							
Number of times recorded	0	0	0	0	0	15	—
Number of animals affected	—	—	—	—	—	12	<0.0001
Trembling							
Number of times recorded	0	0	0	0	0	6	—
Number of animals affected	—	—	—	—	—	5	<0.0001
Ventral recumbency							
Number of times recorded	0	0	0	0	0	11	—
Number of animals affected	—	—	—	—	—	9	<0.0001
External Appearance							
Limb function impaired							
Number of times recorded	0	0	0	0	0	62	—
Number of animals affected	—	—	—	—	—	10	<0.0001
Material around eyes, red							
Number of times recorded	0	0	0	0	0	4	—
Number of animals affected	—	—	—	—	—	3	<0.001
Material around nose, red							
Number of times recorded	0	0	1	0	0	0	—
Number of animals affected	—	—	1	—	—	—	ns
Pelage/Skin							
Hair, sparse							
Number of times recorded	0	0	0	2	3	0	—
Number of animals affected	—	—	—	1	1	—	ns
Hair, wet							
Number of times recorded	0	0	0	0	0	6	—
Number of animals affected	—	—	—	—	—	6	<0.0001
Scabbed area							
Number of times recorded	0	0	1	0	0	0	—
Number of animals affected	—	—	1	—	—	—	ns

We also assessed whether REL-1017 affected the body weight of treated animals. As shown in Table 3, a slight decreased in body weight was observed in animals receiving the positive control (MK-801) at 5 and 2 mg/kg/day and in females receiving REL-1017.

**TABLE 3:** Body weight of animals during the study.

	REL-1017				Methadone	MK-801
Dose (mg/kg/day)	0	20	40	80	20	2
Female^a^						
Day 1	236.6 ± 10.89 (N = 15)	231.3 ± 13.66 (N = 15)	239.7 ± 10.65 (N = 15)	238.7 ± 10.12 (N = 15)	237.8 ± 9.80 (N = 15)	236.0 ± 21.30 (N = 15)
Day 1 (sacrifice)	237.7 ± 12.27 (N = 5)	230.5 ± 13.68 (N = 5)	241.7 ± 12.97 (N = 5)	236.1 ± 9.93 (N = 5)	240.1 ± 8.9 (N = 5)	232.5 ± 21.80 (N = 5)
Day 3 (sacrifice)	223.0 ± 9.70 (N = 5)	220.6 ± 8.91 (N = 5)	227.0 ± 6.28 (N = 5)	216.8 ± 4.49 (N = 5)	230.6 ± 9.81 (N = 5)	219.0 ± 25.60 (N = 5)
Day 5 (sacrifice)	229.4 ± 15.13 (N = 5)	221.6 ± 23.64 (N = 5)	230.6 ± 19.11 (N = 5)	218.4 ± 9.40 (N = 5)	229.0 ± 2.94 (N = 4)	218.6 ± 16.26 (N = 5)
Dose (mg/kg/day) Male^a^	0	31.25	62.5	110	31.25	5
Day 1	346.4 ± 14.03 (N = 15)	344.7 ± 14.06 (N = 15)	342.5 ± 10.93 (N = 15)	351.3 ± 16.23 (N = 15)	341.1 ± 15.38 (N = 15)	341.7 ± 16.55 (N = 15)
Day 1 (sacrifice)	350.0 ± 14.81 (N = 5)	347.9 ± 14.07 (N = 5)	344.9 ± 12.57 (N = 5)	353.3 ± 18.09 (N = 5)	350.9 ± 16.12 (N = 5)	334.4 ± 15.55 (N = 5)
Day 3 (sacrifice)	342.2 ± 12.4 (N = 5)	328.4 ± 15.76 (N = 5)	332.6 ± 7.23 (N = 5)	333.0 ± 16.99 (N = 5)	325.6 ± 17.60 (N = 5)	318.4 ± 14.69 (N = 5)
Day 5 (sacrifice)	353.0 ± 20.46 (N = 5)	349.4 ± 15.88 (N = 5)	352.0 ± 14.30 (N = 5)	345.0 ± 12.91 (N = 4)	340.8 ± 8.73 (N = 4)	327.2 ± 18.46 (N = 5)

a Data are expressed in grams (mean ± SD).

### REL-1017 did Not Cause Transient Pathomorphological Changes to Cortical Neurons

**FIGURE 2:**
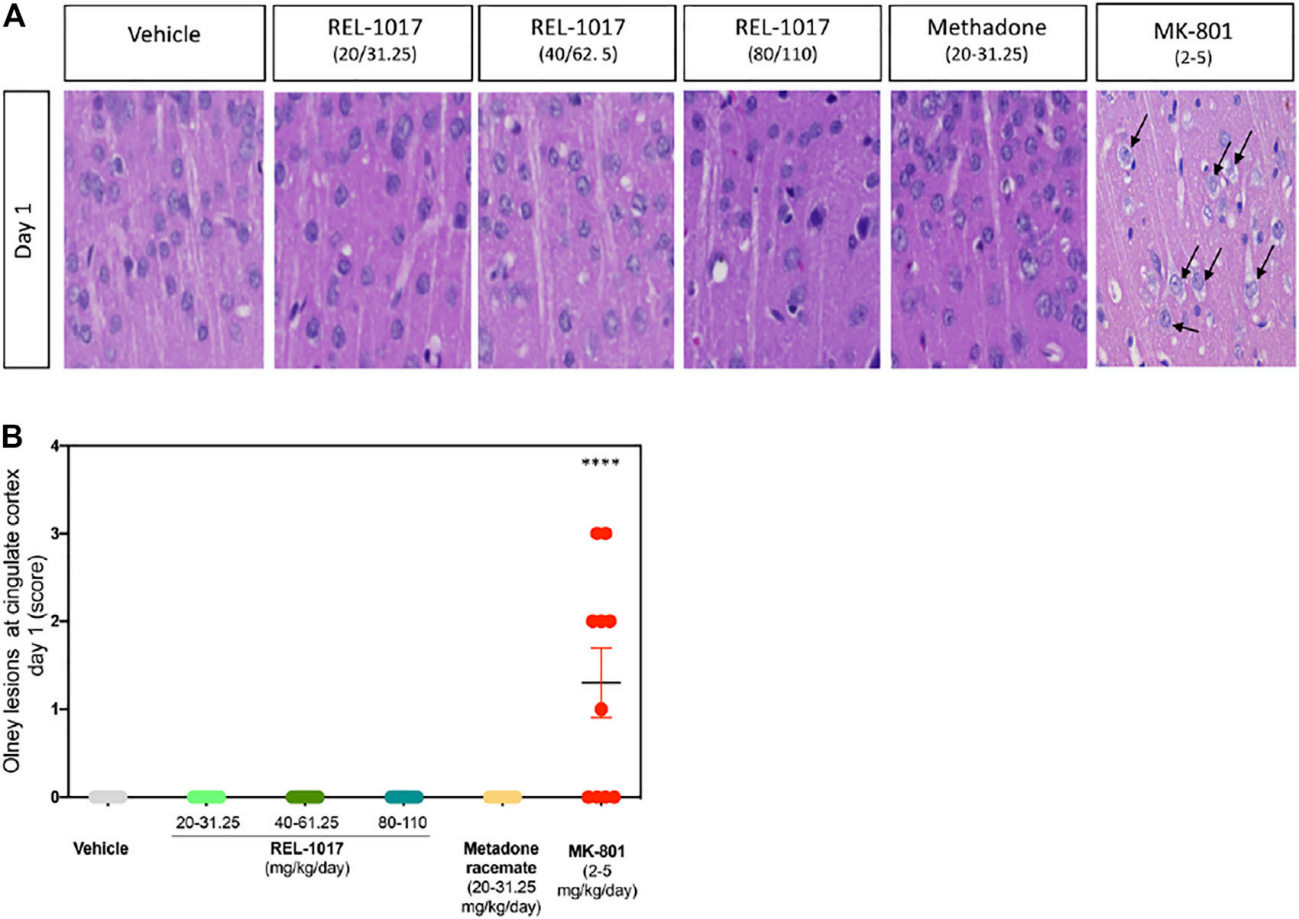
Acute administration of REL-1017 did not cause Olney lesions in the brain cingulate cortex. **(A)** histological examination of the cingulate cortex, one of the brain regions which are most vulnerable to the neurotoxic effect of acute NMDA receptor antagonism. Black arrow marks indicate the presence of the neuronal vacuolations, which are the microscopic features of Olney’s lesions, in the brains of animals treated with MK-801 (positive control). **(B)** To statistically compare differences between treatment groups, we have quantified the number and scored a grade of Olney’s lesion severity. This analysis confirmed that oral gavage administration of REL-1017 in all tested doses to both male and female prague-Dawley rats did not produce Olney’s lesions. Compared to REL-1017, the effect of MK-801 on cortical neurons was statistically significantly different (*p* < 0.0001). Also, the oral administration of methadone racemate to both male and female Sprague-Dawley rats did not produce Olney’s lesions.

In 1989, the neuropathological changes consisting of the formation of multiple vacuoles of heterogeneous size occupying the cytoplasmic compartment (vacuolization of neuronal cytoplasm) in the posterior cingulate and retrosplenial neocortices following acute exposure to NMDAR channel blockers were described (Olney et al., 1989). In the current study, five animals/sex/treatment were sacrificed eight hours following administration of vehicle, or REL-1017 at different doses: low (20–31.25 mg/kg/day, female and male, respectively), medium (40–62.5 mg/kg/day female and male, respectively), or high (80–110 kg/mg, female and male, respectively), or racemic methadone at 31.25 mg/kg/day, or MK-801 at 2–5 mg/kg female and male respectively. Rat brains were analyzed for the presence of neuronal vacuoles (Olney’s effect) by light microscopy on H&E stained brain sections. As shown in Figure 2, Olney lesions, indicative of NMDA receptor antagonist neurotoxicity, were present only in the positive control group MK-801 (6/10, 60% animals). We did not observe Olney lesions in the vehicle, REL-1017 at any dose, or racemic methadone (*p* = ns between REL-1017 at any dose and the vehicle; *p* < 0.001 between MK-801 and all other experimental groups). We scored Onley lesions according to their severity, (see M&M), and found that MK-801 produced mild/moderate (grade 2–3) vacuolation severity with foci of vacuolated neurons forming more diffuse groups of affected cells.

### REL-1017 Did Not Cause Permanent Pathomorphological Changes to Cortical Neurons

**FIGURE 3:**
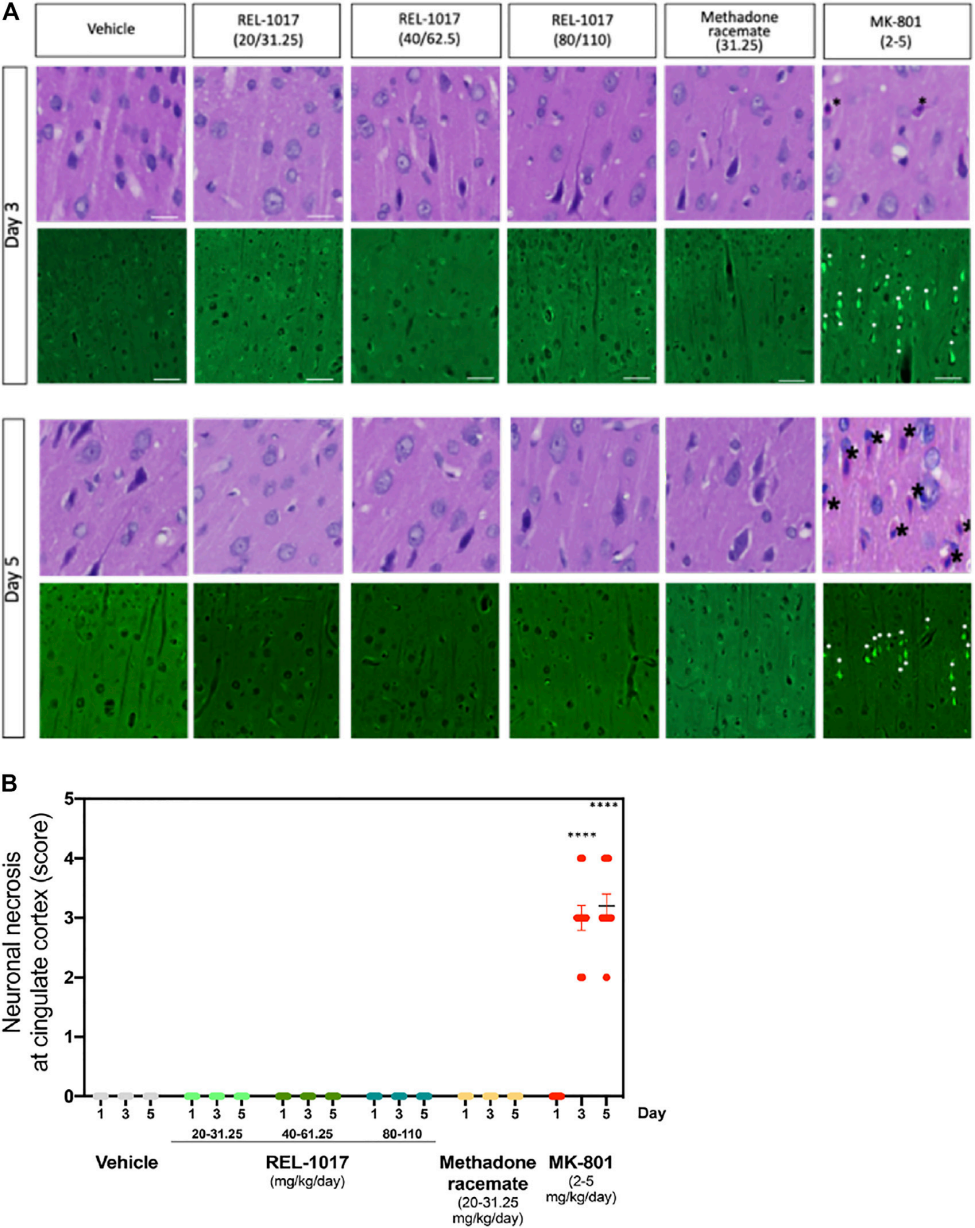
Administration of REL-1017 did not cause neuronal necrosis in brain cingulate cortex. **(A)** Upper row: histological examination (H&E) of the cingulate cortex at Day 3 and 5. Black asterisk marks indicate the presence of necrotic cells, appearing as shrunken, angular cells with pyknotic nuclei and bright eosinophilic cytoplasm, in the brains of animals treated with MK-801 (positive control). Lower row: representative photographs of Fluoro Jade B immunofluorescence, which is a marker of cellular necrosis. Necrosis was present in the cingulate cortex and piriform lobe of MK-801 treated rats on Day 3 and 5 in 100% of the rats (M/F) (white arrowheads indicate some necrotic neuronal nuclei). Consistent with the histological analysis, Fluoro Jade B immunofluorescence was absent in vehicle, REL-1017, or racemic methadone treated rats. **(B)** Quantification of the severity of neuronal necrosis showed a statistical increase in cell necrosis in MK-801 treated brains (*p* < 0.0001 versus all the other groups on Day 3 and 5). Necrotic findings were classified as moderate to marked (ranging from 5 to 40% of necrotic cells).

It has been previously shown that a high dose (2–5 mg/kg) of MK-801 induces neuronal necrosis in the posterior cingulate/retrosplenial cortex of adult rats (Fix et al., 1993). We assessed whether administration of the new NMDAR channel blocker REL-1017 at different doses could cause any neuronal death. We first analyzed brain sections stained by H&E at Day 1, 3, and 5 after different doses administrations. As previously shown, we also observed in MK-801 treated animals the presence of necrotic neurons in the posterior cingulate/retrosplenial cortex, appearing as shrunken, angular cells with pyknotic nuclei and bright eosinophilic cytoplasm (Figure 3A, black asterisk).

Importantly, as shown in Figures 3A, B, no signs of neuronal necrosis were found in the brains of animals treated with vehicle, REL-1017 at any dose, and racemic methadone (*p* = ns). To further confirm and extend these morphological data, we employed Fluoro-Jade B, which is a specific and sensitive fluorescence staining for necrotic neurons. This anionic fluorescein derivative increases the signal to background ratio, thus improving both the contrast and the resolution of the staining of neurons undergoing degeneration. Fluoro-Jade B stained necrotic neurons were detected in the posterior cingulate/retrosplenial cortex of animals treated with MK-801 (white asterisks, Figure 3A). In contrast, no Fluoro-Jade B positive necrotic neurons were detected in any animal from the control, REL-1017, or methadone groups (*p* = ns between REL-1017 at any doses and vehicle). Quantification of the degree and severity of necrosis found in the posterior cingulate/retrosplenial cortex indicated that all MK-801 treated animals had necrosis (100%, *p* < 0.0001 versus all other groups on both Day 3 and 5; Figure 3B). The mean severity of the necrosis of each sample was assessed by scoring the degree of necrosis and was moderate to marked (ranging from 5 to 40% of necrotic cells) in the posterior cingulate/retrosplenial cortex of animals treated with MK-801.

Although less severely, NMDAR antagonists have also been shown to induce irreversible neuronal degeneration in other brain regions, including the olfactory bulb (Newcomer et al., 2000). Therefore, we assessed whether administration of the new NMDAR channel blocker REL-1017 at different doses caused any neuronal death in this region. We confirmed the presence of necrosis in the olfactory bulb neurons of 80% of the rats treated with MK-801. The severity of the neuronal necrosis in this region was scored as minimal (81%) or mild (19%) (Figure S1). Similar to vehicle, REL-1017 did not trigger any necrosis of the olfactory bulb neurons (*p* = ns between REL-1017 at any doses and vehicle).

**FIGURE 4:**
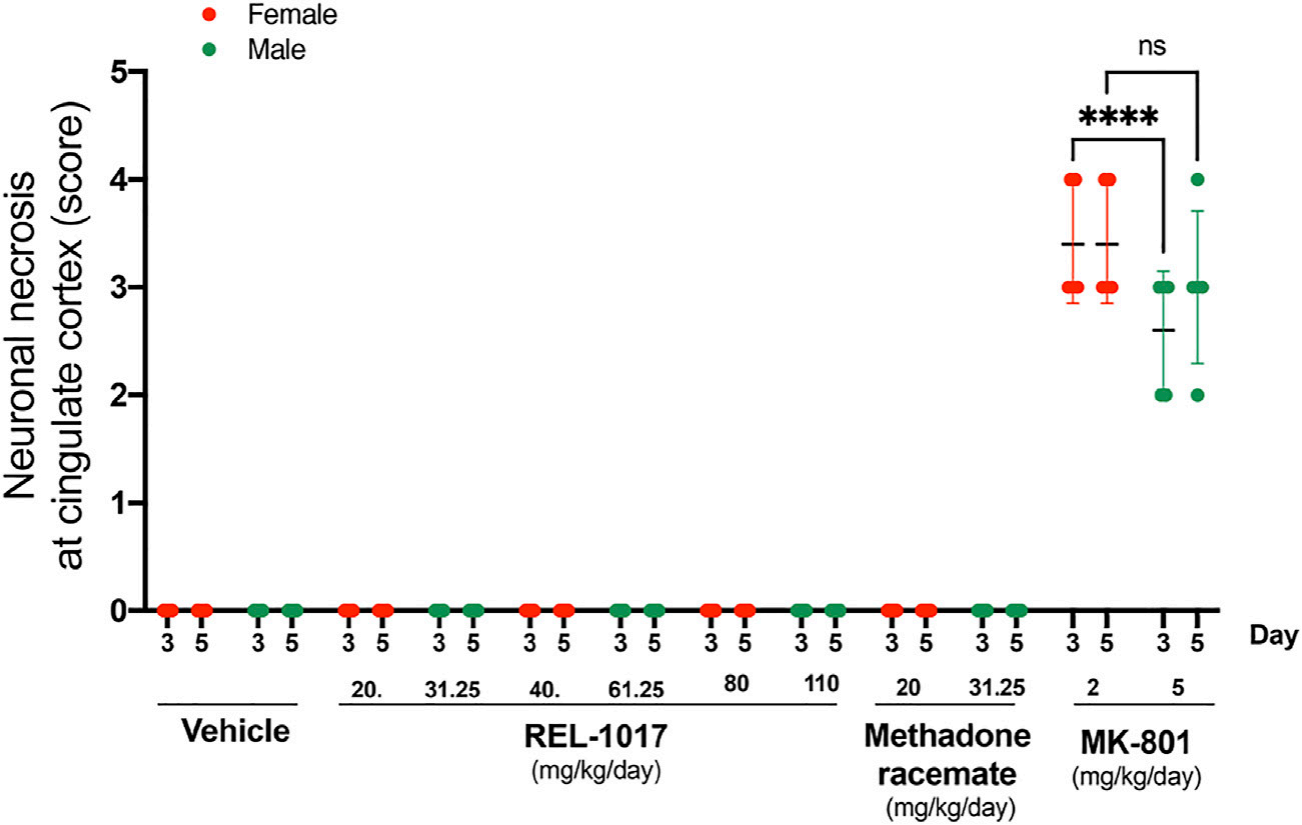
No sex dysmorphism was observed in the REL-1017 effect on cingulate neuronal necrosis. Separate quantification of neuronal necrosis in female and male groups. No differences in neuronal necrosis quantification were observed between female and male REL-1017 treated rats. As expected, we observed a major severity of necrosis in female rats treated with MK-801 (*p* < 0.0001 at Day 3).

We confirmed (Figure 4) the sexual dimorphism of neurotoxic MK-801 effects on neuronal necrosis previously demonstrated by others (Auer, 1996; D'Souza et al., 2002; Honack and Loscher, 1993; Hur et al., 1999). MK-801 triggered more severe neuronal necrosis in female rats (Figure 4, *p* < 0.001 at Day 3). Similarly to the vehicle, there were no lesions in REL-1017 groups of female and male rats (Figure 4).

### REL-1017 Did Not Cause Microgliosis

**FIGURE 5:**
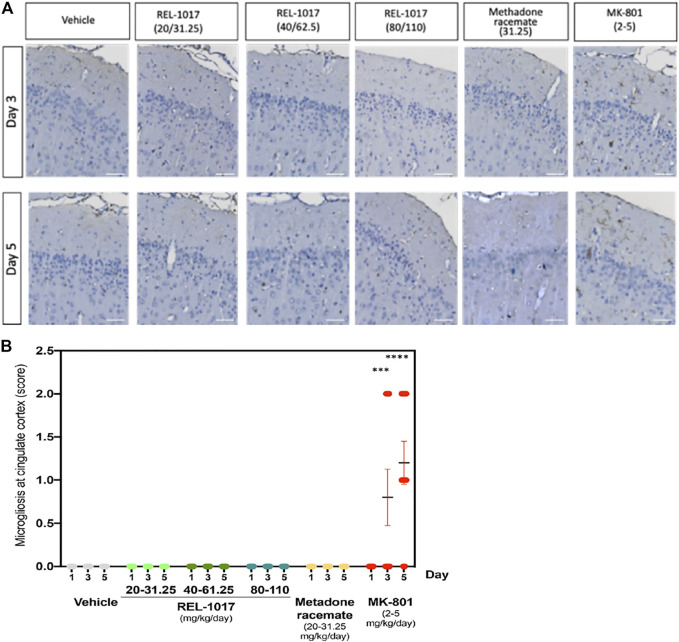
Administration of REL-1017 did not cause microgliosis in the brain cingulate cortex. **(A)** Immunohistochemistry analysis of IBA1 positive (brown) microglial cells in the cingulate cortex at Day 3 and 5. MK-801 treated samples showed a clear increase in IBA1 positive (brown) microglial cells. **(B)** Quantification of microgliosis. The oral administration of REL-1017 at any dose (low, mid, and high) and of methadone racemate to both male and female Sprague-Dawley rats did not induce microgliosis (*p* = ns versus all other groups). Statistical increase in microglia cell number, sored as mild microgliosis, were observed in animals treated with MK-801 at Day 3 (*p* < 0.0001) and 5 (*p* < 0.0001) compared to all the other groups.

Necrotic posterior cingulate/retrosplenial cortical neurons, which are present after 3–5 days following single doses of MK-801, have been shown to be associated with an inflammatory response involving microglia (Harry and Kraft, 2008). To further assess the effect of the NMDAR channel blocker REL-1017, we evaluated and quantified the presence of IBA1 positive microglia in the posterior cingulate/retrosplenial cortical region of treated rats. As expected following the increase in neuronal necrosis, we found a statistical increase of IBA positive microglia cells in the posterior cingulate/retrosplenial cortex after 3 and 5 days of MK-801 treatment (Figure 5A). As shown in Figure 5B, mild microgliosis was observed in 40 and 80% of brains treated with MK-801 on Day 3 and 5, respectively (*p* < 0.001 versus all other groups). Consistent with the absence of neuronal necrosis, no increases in microglia cell number were observed in animals treated with any dose of REL-1017 and racemic methadone (*p* = ns).

## Discussion

In a double-blind, placebo-controlled Phase 2 study, REL-1017 demonstrated rapid, robust, and sustained therapeutic effects in patients with MDDs (Fava et al., 2022).The rapid antidepressant activity of REL-1017 is thought to be due to NMDAR uncompetitive channel block (Gorman et al., 1987; Bettini et al., 2021a) *via* downstream BDNF- and mToR-dependent mechanisms (Fogaca et al., 2019; De Martin et al., 2021).

Following acute exposure to NMDAR channel blockers, several studies described dose- and time-dependent, reversible or irreversible neuropathological changes in cortical neurons. Typically, low doses of an NMDAR antagonist may trigger reversible pathomorphological changes consisting of multiple vacuoles of heterogeneous size, including mitochondria and endoplasmic reticulum (vacuolization of neuronal cytoplasm) in the cingulate cortices (Olney et al., 1989). High dose, or more prolonged NMDAR antagonist treatment caused irreversible neurodegeneration affecting not only neurons of cingulate cortices but also of other cortical regions, including the olfactory bulb (Olney et al., 1991; Fix et al., 1993; Ellison, 1994; Horvath et al., 1997; Wozniak et al., 1998; Newcomer et al., 2000). We, therefore, assessed any potential initial or cumulative neurotoxic effects induced by oral administration of REL-1017 at three different doses: low (20 and 31.25 mg/kg/day), medium (40 and 62.5 mg/kg/day) and high (80 and 110 mg/kg/day) in female and male rats respectively. The effective dose in patients with MDD is 25 mg daily, approximately 0.4 mg/kg/day (Fava et al., 2022).

In REL-1017 treated rats, we did not observe early Olney lesions, which usually appear one day after MK-801 treatment. Similarly, REL-1017 treated rats did not show necrotic neurons both at the cingulate and olfactory bulb cortex at later time points (Day 3 and 5). This effect is statistically different from what was observed in cortical neurons by using MK-801 (Figure 2). Additionally, in contrast with MK-801 treated rats, REL-1017 treated rats did not show evidence of impaired behavior. The molecular mechanisms involved in the neurotoxic mechanism for uncompetitive NMDAR-antagonists are still unclear. GABAα agonists or muscarinic cholinergic antagonists appeared to inhibit the neurotoxic effect of MK801, suggesting that NMDAR block inhibits the action of the GABAergic neurons, impairing their negative modulation on postsynaptic cholinergic or glutamatergic neurons (Olney et al., 1991). In addition to this mechanism, several other transmitter receptor system alterations have been described (Farber et al., 1993; Farber et al., 1995; Farber et al., 1998). MK-801 is a potent uncompetitive NMDAR antagonist that binds preferentially to NMDARs in the open conformation, therefore blocking the channel in a use- and voltage-dependent manner (Huettner and Bean, 1988). REL-1017 blocks NMDAR channel in open conformation with low affinity and low potency (Gorman et al., 1997; Bettini et al., 2021a). In the presence of physiological Mg^2+^ concentrations, REL-1017 preferentially blocks Ca^2+^ currents in GluN1-GluN2D receptor subtypes in a voltage-dependent manner (Bettini et al., 2021b). REL-1017 showed trapping characteristic at the NMDAR NR1-NR2C subtype which is comparable to that of ketamine (Bettini et al., 2021c). Furthermore, in silico studies aimed to understand the structure-activity relationship of REL-1017 and ketamine, suggest that REL-1017, compared to ketamine, displayed differences in binding to and undocking from the NMDAR channel (Bettini et al. unpublished observations). It has been suggested that trapped channel blockers produce NMDAR tonic block rather than phasic block (Mealing et al., 2001). The relatively low NMDAR affinity and low potency of REL-1017 compared to MK-801 and other NMDAR channel blockers and its relatively high, ketamine-like, trapping could explain its robust, rapid, and sustained antidepressant effects in the absence of psychotomimetic effects (Fava et al., 2022) together with the lack of any neuropathological change in cortical neurons as seen in this study.

Previous studies showed different neurotoxic effects of ketamine at high doses (40 mg/kg subcutaneously and up to 60 mg/kg intraperitoneal), possibly due to the use of different rat strains (Onley et al., 1989; Morris et al., 2021). In Onley et al., neurotoxic effects of ketamine were observed in female Sprague-Dawley rats, while in Morris et al., no neurotoxic effects of ketamine were observed up to 60 mg/kg intraperitoneal in Han Wistar rats. However, in both studies, dizocilpine (MK-801) consistently produced neurotoxic effects. Therefore, we decided to employ MK-801 as a positive control, neurotoxic agent. The neurotoxic effect of MK801 is sexually dimorphic since females appear to be more sensitive than males (Auer, 1996; D'Souza et al., 2002; Honack and Loscher, 1993; Hur et al., 1999, Current Study). Our experiments were designed in order to assess any differences between the sexes in the potential neurotoxic effects of REL-1017. The experimental data showed no sex dimorphism in the absence of neurotoxicity of REL-1017, even though female rats are known to have higher REL-1017 plasma concentrations at a given dose than males. The REL-1017 top doses tested in the current study are at or near the maximum tolerated dose for the molecule. This is particularly relevant, since epidemiological studies have shown that the lifetime prevalence of MDD in women is almost twice that in men (Global Burden of Disease Study, 2015). In this study, even the lowest dose employed in both male and female rats was approximately 100- to 500-fold higher than the therapeutic dose employed in humans (25–50 mg/day; Fava et al., 2022). The safety and tolerability of REL-1017 therapy was demonstrated in Phase 1, double-blind, randomized, placebo-controlled, single- and multiple-ascending dose studies in healthy opioid-naive volunteers (Bernstein et al., 2019). REL-1017 has been recently shown to increase brain-derived neurotrophic factor (BDNF) plasma levels in healthy volunteers in a Phase 1 trial (De Martin et al., 2021), suggesting an additional important potential mechanism of neuroprotection.

In conclusion, these results indicate that REL-1017 did not produce initial or cumulative neurotoxic effects or other evidence of damage to cortical neurons, further encouraging the development of REL-1017 as a safe and effective candidate for the rapid treatment of MDD.

## Data Availability

The original contributions presented in the study are included in the article/Supplementary Material, further inquiries can be directed to the corresponding author.
