# Supplementary figures and images for: REL-1017 (Esmethadone), A Novel NMDAR Blocker for the Treatment of MDD is Not Neurotoxic in Sprague-Dawley Rats
Source: Front Pharmacol. 2022 Apr 25;13:863959. doi: 10.3389/fphar.2022.863959 (PMC9097919; doi:10.3389/fphar.2022.863959)

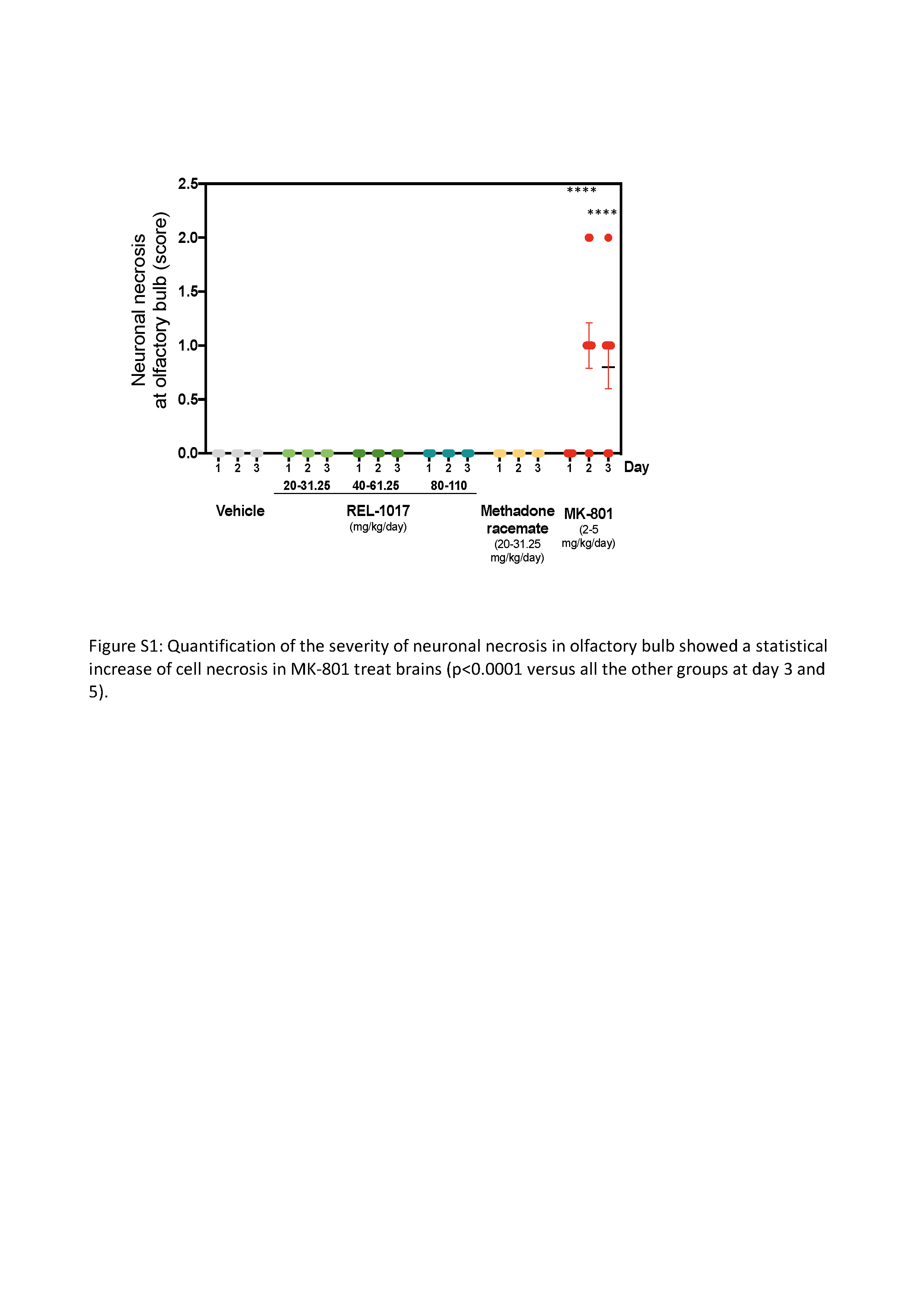

Supplement: Supplementary file 1 [file Image1.tiff]
